# How a Medically Tailored Meal Intervention with Intensive Nutrition Counseling Created Active Coping with Behavior Change for Vulnerable Patients with Lung Cancer

**DOI:** 10.21203/rs.3.rs-3915333/v1

**Published:** 2024-02-02

**Authors:** Pamela Rothpletz-Puglia, Jade Smith, Chloe Pavuk, Jana Leotta, Kimberli Pike, Carolyn J. Presley, Jessica Krok-Schoen, Ashlea Braun, Mary Kathryn Cohen, Gail T Rogers, Ho Kenneth Chui Kwan, Fang Fang Zhang, Colleen Spees

**Affiliations:** Rutgers, The State University of New Jersey; The Ohio State University; The Ohio State University; The Ohio State University; Rutgers, The State University of New Jersey; The Ohio State University Comprehensive Cancer Center; The Ohio State University; Oklahoma State University; Tufts University; Tufts University; Tufts University; Tufts University; The Ohio State University

**Keywords:** food is medicine, lung cancer, nutrition intervention, motivational interviewing, nutrition security, active coping, post-traumatic growth, medically tailored meals, medical nutrition therapy, quality of life

## Abstract

**Purpose:**

The purpose of this study was to assess participants’ perceptions and experiences while participating in a Food is Medicine medically tailored meal plus nutrition counseling intervention to create a theoretical explanation about *how* the intervention worked.

**Methods:**

This interpretive qualitative study included the use of semi-structured interviews with active intervention participants. Purposeful sampling included vulnerable (uninsured, rural zip code residency, racial/ethnic minority, 65 years old, and/or low-income) individuals with lung cancer treated at four cancer centers across the United States. Interviews were recorded, transcribed verbatim, and analyzed using conventional content analysis with principles of grounded theory.

**Results:**

Twenty individuals participated. Data analysis resulted in a theoretical explanation of the intervention’s mechanism of action. The explanatory process includes 3 linked and propositional categories leading to patient resilience: engaging in treatment, adjusting to diagnosis, and active coping. The medically tailored meals plus intensive nutrition counseling engaged participants throughout treatment, which helped participants adjust to their diagnosis, leading to active coping through intentional self-care, behavior change, and improved quality of life.

**Conclusions:**

These findings provide evidence that a food is medicine intervention may buffer some of the adversity related to the diagnosis of lung cancer and create a pathway for participants to experience post-traumatic growth, develop resilience, and change behaviors to actively cope with lung cancer. Medically tailored meals plus intensive nutrition counseling informed by motivational interviewing supported individuals’ adjustment to their diagnosis and resulted in perceived positive behavior change.

## Introduction

Lung cancer affects 1 in 15 men and 1 in 17 women annually and is the leading cause of all cancer-related deaths (25%) [1]. An estimated 80% of people undergoing treatment for lung cancer experience malnutrition, which is characterized by rapid, involuntary weight loss, muscle wasting, and decreased immune function [1, 2]. Individuals presenting with malnutrition often experience greater toxicities to cancer treatment, more frequent hospital admissions, extended length of hospital stays, lower quality of life, and increased mortality [1, 3]. Cancer-related malnutrition is often attributed to the metabolic burden of cancer and side effects of treatments which can lead to nutrition-impact symptoms including fatigue, anorexia, constipation, nausea, diarrhea, mucositis, and gastrointestinal distress [2]. The risk of experiencing malnutrition is compounded when the individual is from a medically underserved population (uninsured, rural resident, racial/ethnic minority, ≥65 years old, and/or low-income) and/or experiencing nutrition and/or food insecurity [4, 5].

Oncology dietitians are specifically trained to address nutrition impact symptoms and mitigate risks for malnutrition, but fewer than 60% of people undergoing treatment for cancer receive any nutrition services [3, 6]. Access to oncology dietitians is limited in the United States (U.S.) due to inconsistent standardized malnutrition screening and lack of reimbursement for nutrition care from Medicaid and Medicare Services and other insurers [3, 7]. With limited insurance reimbursement available, there remains inadequate oncology dietitian staffing in outpatient cancer centers, where 90% of individuals affected by cancer receive treatment [3]. Indeed, the current ratio of dietitians to outpatient patients with cancer across the U.S. is one dietitian to every 2,308 patients [3].

Food is Medicine efforts are underway to improve access to nutrition care and address the root causes of malnutrition among individuals with cancer [8]. Specifically, the provision of home-delivered medically tailored meals, has been shown to reduce nutrition and food insecurity and improve quality of life among people receiving treatment for metastatic cancer [9, 10]. Likewise, intensive nutrition counseling, delivered by dietitians, has been successful in mitigating nutrition impact symptoms and reducing malnutrition risk and severity [6, 11]. Medical nutrition therapy enhanced via the use of motivational interviewing has been identified as an effective intervention for modifiable lifestyle behavior change among individuals experiencing cancer [12]. This enhanced counseling approach is a technique that provides a person-centered supportive environment conducive to mutual trust, engagement, and goal setting.

*NutriCare* is the first medically tailored meals plus intensive nutrition counseling intervention in the Food is Medicine space designed to fully integrate food and nutrition into oncology care for vulnerable patients with lung cancer. This multi-center randomized controlled clinical trial (NCT04986670) is provided throughout active cancer treatment and into post active treatment survivorship. Preliminary findings from this study documented improvements in diet quality, food security, and quality of life in the intervention group as compared to controls.

To learn more in-depth about participant perceptions and experiences of the NutriCare intervention, the research team developed a phase 2 qualitative study. The aim was to assess how the NutriCare intervention worked to create a theoretical explanation to guide future interventions. Theoretical explanations are based on patterned relationships between concepts that include propositional logic explaining why something may happen in a similar context [13].

## Methods

### Study Design

This phase 2 qualitative inquiry was based in pragmatism where participants perceptions about useful aspects and applications of an intervention were generated from in-depth interviews [14]. Principles of grounded theory were implemented within conventional content analysis of the interview transcripts to generate evaluative categories (content analysis) [15] and theory about how the intervention worked (grounded theory) [16]. A full-fledged grounded theory study was not conducted since the theoretical framework was generated post-interviews and the process did not include iterative data collection to delve further into the interpretations [17]. A pragmatic qualitative approach enabled the flexibility to incorporate principles of grounded theory with the content analysis since content analysis and grounded theory are based in constructivism and are methodologically congruent [18].

#### Ethical approval

The protocol was approved by the Institutional Review Boards of Tufts University (#00002515) and adheres to the principles of the Declaration of Helsinki. All participants were informed of the purpose and procedures of the study. Verbal consent was obtained from all participants prior to data collection.

#### Participants and recruitment

Phase 1 of the NutriCare study enrolled 158 individuals recently diagnosed with lung cancer from four medical centers nationwide: Tufts Medical Center, MD Anderson Cancer Center, The Ohio State University Comprehensive Cancer Center, and Fox Chase Cancer Center. All participants met one or more of the following United States Health Resources and Services Administration vulnerability criteria: uninsured, rural residency, racial/ethnic minority, 65 years old, and/or low-income [4].

Twenty study participants were purposefully sampled for lung cancer diagnosis, vulnerability criteria, and inclusion criteria [39].

During week eight of the participant’s nutrition counseling session, the study dietitian assessed the participants’ willingness to participate in an interview. This time frame corresponded with the end of the first and most intensive phase of the study. At this point in the study, NutriCare participants had received six to eight weeks of both medically tailored meals plus intensive nutrition counseling. If a patient agreed to participate, the dietitian scheduled the interview between the participant and a trained graduate student researcher.

#### Pre-Interview Training

Prior to beginning data collection, two graduate student researchers completed three 1-hour qualitative research training sessions led by research team members with expertise in motivational interviewing (JS) and qualitative research (PRP). Each of the two graduate students (CP, JL) conducted a pilot semi-structured interview with a NutriCare participant and an experienced interviewer mentor (PRP). Pilot interviews were used to inform and modify the interview guide and to provide the graduate students with feedback on interviewing techniques.

#### Data collection

Semi-structured, telephonic interviews were conducted by graduate student researchers between August 2021 and February 2022. Probes within the interview guide (Table 1) allowed for flexibility in participant responses. Interviewers kept a reflexivity memo of thoughts, perceptions, and personal assumptions after each interview experience and these experiences were discussed during regular team meetings.

#### Data analysis

Interviews were audio-recorded, transcribed verbatim, and checked for accuracy to ensure an exact account of participant responses. Transcripts were anonymized and then analyzed using conventional content analysis and with principles of constructivist grounded theory [15–17]. The analysis began with 5 research team members each creating a conceptual memo for each of the first 9 interview transcripts. The conceptual memos each team member created led to team discussions and consensus on initial concepts and the development of a preliminary codebook. The interview transcripts were then loaded into NVivo12^™^ for first level, line-by-line coding (i.e., labeling concepts) by the graduate student researchers. The coding was assessed at >80% agreement among the two analysts. After consensus was reached on the discrepant coding, the graduate students double-coded 11 more transcripts. The research team met regularly to discuss and compare the codes to generate tentative categories of conceptually linked concepts. Once the team achieved consensus on the categories generated, the categories were compared within and between participant transcripts for identification of patterns, divergence and linkages for development of a propositional logic to develop an explanatory process for the NutriCare intervention [19]. Recruitment, interviews, and analysis continued until data saturation was met, defined as the absence of new codes.

## Results

A total of n = 20 active NutriCare participants were interviewed between August 2021 through February 2022 and included in the data analysis. Three additional participants consented to the study but did not answer the phone for their scheduled interview. Data saturation was determined after 17 interviews, but 3 additional interviews were conducted to ensure sufficient data for thick and thorough descriptions to be applicable in other contexts (i.e., transferability) [20]. The duration of the interviews ranged from 14 to 64 minutes with an average interview length of 30 minutes.

### Participant Demographics and Clinical Characteristics

Fifty-five percent of the interviewed participants had stage IV lung cancer (n = 11), 6 participants had stage III lung cancer, 1 participant had stage II, 1 participant had stage I, and 1 participant was classified as limited stage small cell lung cancer, in which malignancy was contained to one lung and treatable. Of the twenty participants, 60% were female (n = 13), 85% (n = 17) were white, and 55% (n = 11) were ≥ 65 years old. Forty percent (n = 8) of participants were rural residents, 100% (n = 20) had health insurance, 45% (n = 9) were on Medicare, 85% (n = 17) reported to be at or above 130% of the poverty line, and two participants declined to answer this question (Table 1).

### Explanatory Theoretical Process Framework for the NutriCare Intervention

The categories and propositional logic generated from the content analysis with principles of grounded theory served to construct the analytic findings. These findings include an explanatory process framework comprised of three propositional categories generated from the data that describe how the NutriCare intervention worked:

1. Engaging in treatment: The medically tailored meals plus intensive nutrition counseling informed by motivational interviewing catalyzed participants engagement in their own self-care because it was patient-centered, socially supportive, challenging, and empowering.
2. Adjusting to diagnosis: Engagement in the intervention helped participants adjust to their diagnosis because the meals were convenient and provided food security and the support created the capacity for participants to adjust.
3. Active coping with health behavior: Participants’ adjustment to their diagnoses enabled learning, active coping, and post-traumatic growth during the intervention.

This process led to the overarching finding that the NutriCare intervention provided a pathway for participants to develop resilience for actively coping with lung cancer.

### Engaging in Treatment

Participants actively participated in the weekly medically tailored meals and engaged in the intensive nutrition counseling. The drivers of engagement in the NutriCare intervention were related to participant’s perceptions of support, encouragement, empowerment, trust, being challenged, and feeling heard.

The weekly nutrition counseling with motivational interviewing engaged participants partly because the dietitian listened to and supported the participants. Many participants mentioned how the dietitian listened:

“She listens. She listens to things I say. She’s there when, you know — if there’s something on my mind. She listens to — you know — things, or how I feel. And she tries to answer questions for me.” In addition to active listening, participants described the nutrition counseling as personalized. For example, “She is trying to work with me and make suggestions within my stubbornness (laughs) — would be a way to say it. Yeah, I mean…I think [about] what she has shared with me, and what I’ve tried to…incorporate.”

Participants also described feeling encouraged by the dietitian during the nutrition counseling and one participant stated, “She boosts my morale.” Another participant explained:

“At first, I thought, this is ridiculous to call and talk to me about food. I think she’s really done some good by pushing me. She’ll say okay, next, let’s see if you can add a vegetable or two vegetables — so, she’s challenged me. And I guess when somebody challenges me, I’ll take ‘em on it.” This quote exemplifies how the dietitian challenged the participant to engage in care. The goal setting that often occurred also appeared to encourage participants. For example, “She had me drink those little shakes when I get nauseated and keeping something in my stomach. That was it. That was one of the goals. To keep something [in my stomach], eat little meals throughout the day which helped.” The participants also discussed how the dietitian knew when to scale back on the goal setting as demonstrated by this quote, “She sets goals for me every week. We set them together actually. Like, on those weeks I wasn’t feeling good after the radiation, I told her, [dietitian] I really don’t have a goal this week, except for surviving and get through this. And she said, that’s all we need.”

The dietitian provided tailored and person-centered care that engaged participants during all phases of their cancer treatment. For example:

“Yeah so [dietitian] provided lots of tips. I had 30 radiation treatments, and they burned my esophagus, and it has only been healed a few weeks. So you know, eating was hard for a long, long time and there were weeks that that I maybe ate 3 bites a day. It got pretty rough in the end, but when I started feeling better, you know hearing those times, you know encouraging me to eat soft foods and soup. You know, it’s like ‘I really don’t care what you eat health wise. You just gotta eat.’ And in the beginning encouraging lots of protein. I guess getting my body built up for what she knew was coming. And, just you know, since I’ve been feeling better and cooking more trying to help encourage me to eat more vegetables and fruits.”

The medically tailored meals provided a convenient resource to patients making the collaborative nutrition counseling goals and focus achievable. The meals provided a framework patients could use as a model for their dietary pattern. For example:

“I couldn’t eat. So that’s where MANNA came through for me. They had certain things that they had pureed. They have many options for me….things that were more tolerable for me. And financially, that also helped me.” In addition, to providing affordable access to medically tailored meals to meet individualized needs the patients shared that the meals caused them to shift their daily schedules to prioritize their nutrition status. One patient stated: “So NutriCare has set up a system of meals….. before I went on ….. NutriCare, I didn’t eat all day. I just ate one meal at night.”

The participants reported engaging in the NutriCare intervention because they felt heard, supported, and encouraged during the intervention. This approach created patient and provider trust. For example, a participant stated:

“I made a commitment to [oncologist] that she would help me, get me well enough to see my grandson born, which has been seven months ago. Cause she’d work with me, and fight with me, and everything else. I did everything she wanted me to do and ask. I made that commitment and promise to her, so I’ve got to do that. You’ve got to make a commitment to yourself, you can’t just say, well, I think I’m going to try. You’ve got to sit down and commit yourself to the the program.”

The participants’ engagement in the NutriCare intervention seems to be attributed in part to the patient-provider relationships created during the tailored nutrition counseling. The combination of nutrition counseling with motivational interviewing, perceived benefits, trust, and person-centered care empowered participants to engage in self-care in multiple ways including the consumption of the medically tailored meals despite not feeling well.

### Adjustment to Diagnosis

Most participants talked about the difficulty of living with lung cancer. For example, “I’m going through radiation right now and it’s a bad thing, my esophagus’s not okay, and it really, it wears me out terribly I mean I’m just exhausted.” However, the participants perceived the NutriCare intervention as equipping them to handle their illness by providing tips and solutions that created the personal bandwidth to adjust to their lung cancer diagnosis. For instance, many participants talked about the benefits of meal delivery including reduced stress around meal preparation. One participant shared:

“I’m not able to, like, go out or cook and stuff. So, it’s good to know that there’s something in the freezer that I can take out. So, I mean, I know this is going to sound really silly but I like the fact that I really don’t have to think about food shopping.”

Participants talked about how the NutriCare intervention helped them when they are feeling unwell. One participant commented on how the NutriCare intervention helped her adjust by stating “those tips that she’s giving you — been able to — that kinda helps your cooking process. Giving you some ideas of things to cook for yourself or kind of just help you cope with those really tough treatments and diagnosis and things like that.” Several participants discussed the specific benefits of pre- and probiotic foods and nutrition recommendations provided through motivational interviewing and medical nutrition therapy. For example:

“[NutriCare] has completely straightened out my old digestive system, and that was a miserable stage for me in the physical condition that I’m in. And I had wished I owned stock in Imodium I was having to use it so much. So, I have none [diarrhea]. Absolutely none under your food and probiotic pill from [medical center] and [dietitian’s] list of 10 or 12 prebiotic foods to incorporate with that.”

Many of the participants’ comments related to learning new ideas to help them adjust:

“I could sort of put in my toolbox to get me through.” and “I really enjoyed the conversation with [dietitian]. She’s given me really a lot of good ideas and some good recipes.” The new ideas that participants discussed as helping them ranged from tangible nutrition tips to intentional practices for relaxation, stress relief, and physical activity patterns. The participants acknowledged that the health and wellness tips they received improved their quality of life. One participant stated, “I’ve had pretty good quality of life, you know, through the sickness. I mean, I’m stage 4, and uh you know, I’ve had a couple difficulties here and there, but for the most part I’ve got a good quality life. And I think a lot of that is due to my nutrition.” Another patient emphasized the need for consistency provided by the meals stating, “…let’s get the word out there that everybody needs to eat better…have proper nutrition. Try to eat more – no less than three meals a day.”

Upon completion of the most intensive portion of the intervention patients recognized the difference the intervention had made in their ability to improve their health and cope with their diagnosis. Moving them to share what a difference the NutriCare program could have made for had this opportunity been available earlier. For example, two patients shared:

“40, 50 years old they should be thinking about taking care of themselves I mean, I’ve learned that, that’s real. Nutrition is very important. Very important. You don’t realize. More than that, I mean if I had been doing this to begin with I might not have even wound up at the doctor. If you do your body right to start with, you might not have even wound up with this crap.” In addition, to the recognition of the role nutrition plays for cancer prevention patients value the support medically tailored meals and counseling provide during treatment stating, “I wish I would have had this program when I was diagnosed the first time.”

### Active Coping with Health Behavior Change for Self-Care

The NutriCare intervention supported participants to adjust to their diagnosis by helping them overcome nutrition and meal preparation challenges. Participants described succeeding with overcoming some of the nutrition challenges of living with lung cancer by changing health behavior to actively cope and enact self-care. A participant stated:

“I want to prolong life, and if I was to do that, I’d have to be part of the work myself. I can’t rely on drugs and treatments and everything else that I did… I got to know [dietitian] real well… Talking with her, she’s like a godsend as far as, to help me out, give me different ideas when I was feeling sick and what to eat before chemo and after. I mean, I listened to her, and it helped me out tremendously. I mean, I’ve got energy all day now. You see what I’m saying. I’ve made the changes. I’ve actually worked with my children, especially my daughter, and my wife has done the same thing. We have no white bread at home. We went to whole grain everything we can. Same with my daughter. My daughter eats this something called ‘no bread’ that’s ancient grain or something like that. But we’re all trying to make the changes. Plus, she can see how much better it makes me feel.”

The participants described making numerous dietary and lifestyle changes due to the Nutricare intervention. These behavior changes included drinking more water, eating more fruits, vegetables, fiber, and eating less highly processed foods. One participant said:

“I no longer smoke anything. I try not to eat very rarely do I eat red meat…and I try to make sure to eat fruits and vegetables every day. We went shopping today to get more vegetables… I mean not vegetables, more fruit in…cause we were running low on fruit. But it’s worked out…and with my grandkids and everything else, I mean I’ve got fruit here for them also.” Many of the participants talked about how their dietary patterns are much healthier now. For example, a participant shared: “I mean I’m eating salads, I’m eating fruits. I’m eating, you know, I’m eating good food.”

The changes they made were incremental and sometimes described as difficult. For example, a participant stated: “So I figured I’d start learning to change. You know, so that’s what I’m doing, and when you’ve ate one way for 60 some odd years. It’s a challenge to change your way of thinking.” Some participants described how it was difficult to eat differently than the rest of the family. For example, “My husband is still eating southern comfort foods while I’m eating three [medically tailored] meals. And I stay on it religiously. I have stayed on it religiously.”

All the participants made dietary changes and often described how their eating patterns have substantially changed due to NutriCare. One participant said:

“At first it was difficult. I’m not going to say it wasn’t because I was used to eating — maybe something for breakfast — a lot of times not and just eating a large supper. From being — like I said — working 8–10 hours a day, I just didn’t eat. I didn’t eat well, I didn’t eat three meals a day, and I didn’t eat the proper food. I would wait until supper time, and then I’d just basically gorged myself. So, I’d be full through the next day. And the NutriCare, like I say, system working with [dietitian] and everything else has taught me that I have to have a minimum of three meals a day. And then even when I am sick, I just got over pneumonia and had radiation treatment and everything else. I might eat four or five meals a day — smaller meals.”

Another shared:

“What that’s taught me though is that I’ve got nutrition throughout the day, and I have my energy throughout the day. And you know, it’s taught me about eating well balanced food. Like right now — I used to eat a lot of white bread and stuff like that….If I do eat bread, I eat whole wheat or whole grain, but I don’t eat that much bread anymore. I don’t eat that much red meat anymore.” “I’ve got to make some tough decisions. Most of the time they’ve been hard to get used to, but they’ve worked out good. I mean I try to make sure I eat fresh fruit every day. I eat vegetables every day, which I would have never done before…. I may have gone for week and a half before I got sick and not have a vegetable.”

Patients recognized both their own role in the intervention, and the benefits provided to them through counseling.

One patient stated, “ So nutrition is very important it has to be part of your regular everyday life” indicating their understanding that they are responsible for their choices on a daily basis. While also expressing appreciation for the guidance provided through the nutrition counseling as stated here, “ Personally, I think every cancer patient …. should see a dietitian every time they see their doctor because the thing is, if we don’t eat right, we’re not going to have the energy or the right nutrition for our body to fight anything.

In addition to dietary changes, many participants also discussed increasing their physical activity, and time with family, friends, and pets. For example, “I guess with some encouragement from [dietitian]. I go to the Y every morning at eight and exercise in the pool. And then do yoga after that.”

### Resilience

**Figure 1:**
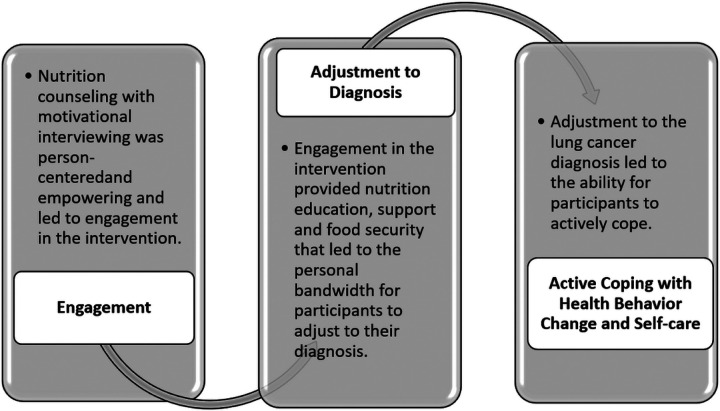
The NutriCare Process

The theoretical process that explained how NutriCare worked is based on the patterned relationships we discerned as engaging participants in treatment, adjustment to diagnosis, and active coping and self-care. NutriCare appeared to work by engaging participants in treatment to support their adjustment to a lung cancer diagnosis and treatment which enabled active coping and self-care (Fig. 1).

The overarching outcome of the NutriCare process was that the intervention appeared to buffer some of the adversity related to a lung cancer diagnosis, and this helped participants develop resilience. This quote exemplifies the resilience participants described, “There are just days you just you get feeling bad and you think, wow, I don’t know if I’m going to fight this any longer or not…but then you decide to do it. And I also think your mental health, a lot of the good nutrition helps. Because you are more alert…sharper.”

Behavior changes that were adopted appear to lead to post-traumatic growth and resilience. Participants reported regarding the intervention as a positive experience, and that it helped them become physically and mentally equipped with tools to help them cope with the lung cancer diagnosis. They perceived the intervention as beneficial and reported feeling better when they acted on the mutually developed goals from the nutrition counseling with motivational interviewing. For example:

“And like I say, [dietitian] through the NutriCare program has taught me different things I can eat and drink to help calm, you know, combat the nausea and you know, just everything else. It’s just made me feel a lot better. The NutriCare and the food from [MANNA] and everything else, it just made it easier for me to prepare, or for [caregiver] to prepare for me when I was really sick. But for the most part, it gave me my dietary intake for that day. And if I eat those three meals, I know I have what was required for me to fight this cancer.” Another patient shared how the program allowed them to execute the behaviors they knew were beneficial even when they were not motivated to do so, thus exhibiting resilience. The patient shared, “Unless it’s been a long time since I’ve eaten, I don’t get hungry. But I know that I need to do it so I sit down and I eat anyway.

## Discussion

The purpose of this study was to explore the perceptions and experiences of individuals enrolled in the intervention phase of the NutriCare study to provide a theoretical explanation for the intervention process. Our findings suggest that home-delivered medically tailored meals and weekly remote nutrition counseling created engagement in treatment which helped with their adjustment to diagnosis leading to active coping and self-care behaviors (Fig. 1).

Most interventions in chronic disease target health behavior change and report on those outcomes, but few elucidate *how* the intervention process worked [21]. Understanding how an intervention worked is important because a theoretical explanation of the process will guide the development of future programs and research. For example, we learned that person-centered motivational interviewing and medically tailored meals are key ingredients for promulgating an adjustment to diagnosis and active coping with self-care.

Furthermore, understanding how this food is medicine intervention worked is significant because a cancer diagnosis has complex psychological and physical impacts [22–24]. Patients report feeling overwhelmed by the complexity of care, feeling ill-prepared to manage side effects of treatment, isolated, unsupported, and “out of their depth” [23]. Despite this, cancer patients engaged in NutriCare and other nutrition and lifestyle interventions report creating personal growth since lifestyle changes are challenges that patients can control at a time of uncertainty [22, 24]. This ability to learn and change despite experiencing adversity is remarkable but aligns with transformative learning and social-cognitive transition models where one is challenged to question existing habits for personal growth [25, 26].

Motivational interviewing integrated with nutrition counseling within the NutriCare intervention, appears to have supported patients to question their lifestyle habits in a way that enabled change. Research shows that motivational interviewing is effective or outperforms traditional educational interventions without motivational interviewing for a broad range of diseases [27–29]. Motivational interviewing begins with developing trust and is predicated on engaging patients, honoring autonomy, identifying intrinsic motivation, and empowering patients to make behavior change to respect their personal values and priorities, akin to shared decision-making. The participants in the NutriCare study engaged in shared decision-making, becoming more purposeful about making lifestyle changes to actively cope with a serious diagnosis leading to post-traumatic growth and resilience.

Post-traumatic growth usually involves some level of increased appreciation for life, self-care, improved health behaviors, and improved perception of personal strength, making life more meaningful [24, 30]. During aggressive cancer treatments, individuals often report feeling overwhelmed by nutrition-related issues such as food availability, complications related to nutrition impact symptoms, and limited, if any, access to nutrition care professionals [23]. These critical gaps in cancer care contribute to the development of malnutrition and associated increased mortality for vulnerable patients [3]. Full integration of food and nutrition into cancer care for vulnerable individuals with lung cancer led to participant perceptions of improvements in dietary patterns and physical activity. The participants described various health behaviors used to prevent or mitigate symptoms. Participants reported that these changes collectively impacted their quality of life positively. These findings underscore the importance of nutrition and the need for dietitians on the oncology care team across the cancer continuum.

NutriCare participants reported that the medically tailored meals also contributed to coping and psychological resilience. Coping is defined as means by which patient participants manage the physical and emotional impacts of their diagnosis [31]. A diagnosis of advanced lung cancer is often accompanied by a myriad of psychological stressors in conjunction with a physical symptom burden, a compromised quality of life, and various monumental medical decisions [32]. Depending on the severity of their diagnosis, people with cancer are faced with the decision to endure a mentally and physically demanding treatment course that often results in symptoms and resulting malnutrition, therefore making nutrition care a critical component in the treatment of advanced lung cancer [32].

Psychological resilience is one’s ability to achieve and/or reestablish a stable mental and physical state in response to a stressful life event [24]. Individual characteristics of psychological resilience include actively coping to facilitate the adjustment to a diagnosis through a positive and optimistic disposition, self-efficacy, cognitive flexibility, social support, and a sense of spirituality [24]. The NutriCare nutrition counseling provided social support and created self-efficacy through shared decision-making. The NutriCare participants communicated that the meal delivery made life easier. This provides preliminary evidence that the meal delivery may have created cognitive flexibility by reducing the stress and cognitive load related to finances, food procurement, and food preparation. Theoretically, this created the emotional bandwidth or cognitive flexibility for participants to actively cope and change lifestyle behaviors [33–35].

### Study Limitations

To ensure trustworthiness, we provided quotes to illustrate interpretations for representational adequacy and credibility, described the findings in sufficient depth to demonstrate data saturation and transferability, reached consensus on data analysis through team discussions for dependability, but we did not seek participant confirmation of the findings for confirmability.[20] The research team was comprised of content and methodological experts, and the graduate students were trained in interviewing and data analysis, but the interviews may have been more in-depth if the interviewers had more experience. As a team, we practiced reflexivity where we discussed our biases and assumptions to guard against influencing the data analysis, but all the team members were registered dietitians in training, or oncologists involved with the NutriCare intervention. Finally, we reached data saturation at interview 17 of 20, but most of the participants were living with stage III or IV non-small cell lung cancer. Our findings may have been more nuanced if we interviewed more participants at an early stage of disease or with small cell lung cancer.

## Conclusion

We developed a theoretical explanation about how the NutriCare intervention worked. This process included three linked and propositional categories: engaging in treatment, adjusting to diagnosis, and active coping— resulting in resilience. The person-centered nutrition counseling with motivational interviewing engaged participants in treatment, which helped participants adjust to their diagnosis, leading to active coping through intentional self-care, behavior change, and improved quality of life.

Our findings provide evidence that a nutrition intervention created a pathway for participants to experience post-traumatic growth. Participants developed resilience and changed health behavior to actively cope with lung cancer. Nutrition counseling with motivational interviewing and meals supported individuals’ adjustment to their diagnosis for positive behavior change. Motivational interviewing in nutrition counseling is shown to be effective for creating lifestyle behavior change and our study adds to this evidence.

In the NutriCare study, nutrition counseling with motivational interviewing and meal delivery appear to mitigate the impact of living with lung cancer by supporting participants to actively cope and make lifestyle behavior changes that created resilience and improved their quality of life. While there is some research about patient’s social cognitive transition in cancer, more research is needed to understand the impact different types and dosages of interventions on patient’s ability to adjust. [26, 36]

## Figures and Tables

**Table 1.** Participant Characteristics (n=20)

Mean age	65.8 (9.5)
Age Categories	
<60	3 (15.0%)
61-<70	9 (45.0%)
70-<80	8 (40.0%)
Patient assigned sex at birth	
Male	7 (35.0%)
Female	13 (65.0%)
Race	
Non-Hispanic White	17 (85.0%)
Non-Hispanic Black	3 (15.0%)
Household income	
$15,000-$19,999	3 (15.0%)
$25,000-$34,999	6 (30.0%)
$45,000 - $54,999	3 (15.0%)
$75,000+	6 (30.0%)
Don’t want to answer	2 (10.0%)
Health insurance	
Yes	20 (100.0%)
Medicare	
Yes	9 (45.0%)
No	10 (50.0%)
Don’t want to answer	1 (5.0%)
Mean BMI	29.2 (7.4)
BMI categories	
Underweight	1 (5.0%)
Normal weight	2 (10.0%)
Overweight	10 (50.0%)
Obese	7 (35.0%)
Have you smoked at least 100 cigarettes in your entire life?	
Yes	18 (90.0%)
No	2 (10.0%)
Number of comorbidities	
No comorbidity	2 (10.0%)
1–2 comorbidities	14 (70.0%)
3–4 comorbidities	4 (20.0%)
Histologic type of lung cancer at diagnosis	
Small cell	2 (10.0%)
Non-small cell	18 (90.0%)
Early or Late-stage	
Early (Small cell limited/Non-small cell stage I-II)	3 (15.0%)
Late (Small cell extensive/Non-small cell stage III-IV)	17 (85.0%)
ECOG	
0	5 (25.0%)
1	13 (65.0%)
2	2 (10.0%)

## Data Availability

The dataset generated and analyzed in the current study are not publicly available due to the sensitive nature of some of the qualitative interview responses, but deidentified data are available from the corresponding author on reasonable request.
